# The Protective Effects of Alisol A 24-Acetate from *Alisma canaliculatum* on Ovariectomy Induced Bone Loss *in Vivo*

**DOI:** 10.3390/molecules21010074

**Published:** 2016-01-09

**Authors:** Yun-Ho Hwang, Kyung-Yun Kang, Sung-Ju Lee, Sang-Jip Nam, Young-Jin Son, Sung-Tae Yee

**Affiliations:** 1 Department of Pharmacy, Sunchon National University, 255 Joongang-Ro, Seokhyeon-Dong, Suncheon 549-742, Korea; hyh7733@naver.com (Y.-H.H.); kang8404@nate.com (K.-Y.K.); 6525140@hanmail.net (S.-J.L.); sony@sunchon.ac.kr (Y.-J.S.); 2 Department of Chemistry and Nano Science, Ewha Womans University, Seoul 120-750, Korea; sjnam@ewha.ac.kr

**Keywords:** osteoporosis, bone loss, alisol A 24-acetate, *Alisma canaliculatum*, regulator T cell, traditional Korean medicine

## Abstract

*Alisma canaliculatum* is a herb commonly used in traditional Korean medicine, and has been shown in scientific studies to have antitumor, diuretic hepatoprotective, and antibacterial effects. Recently, the anti-osteoclastogenesis of alisol A 24-acetate from *Alisma canaliculatum* was investigated *in vitro*. However, the influence of alisol A 24-acetate on osteoporosis in animals has not been investigated. The present study was undertaken to investigate the anti-osteoporotic effect of alisol A 24-acetate on bone mass in ovariectomized (OVX) mice and to identify the mechanism responsible for its effects. OVX mice were treated daily with 0.5 or 2 μg/g of alisol A 24-acetate for a period of six weeks. It was found that these administrations significantly suppressed osteoporosis in OVX mice and improved bone morphometric parameters. The serum estradiol, bone alkaline phosphatase levels, regulatory T/Th17 cell numbers were significantly increased by alisol A 24-acetate as compared with untreated OVX mice. In addition, TRAP activity was inhibited by alisol A 24-acetate in OVX mice. These results suggest alisol A 24-acetate effectively prevents bone loss in OVX mice, and that it can be considered a potential therapeutic for the treatment of postmenopausal osteoporosis.

## 1. Introduction

Bone is a dynamic tissue that undergoes continual adaption during vertebrate life to preserve skeletal size and shape, and to regulate mineral homeostasis. Bone remodeling is the removal and formation of damaged bone to maintain skeletal integrity and mineral homeostasis, whereas bone homeostasis is maintained by a balance between bone-resorbing osteoclasts and bone-forming osteoblasts [1].

Osteoporosis is a common disease among the elderly, and a serious worldwide health problem [2,3]. It causes loss of bone mass and strength and deterioration of bone microarchitecture, which increase the risk of fragility fractures [4]. Osteoporosis especially affects post-menopausal women and is associated with estrogen deficiency, which enhances osteoclast production, and thus, disrupts osteoblast/osteoclast balance [5]. T cells are key inducers of bone loss in the presence of estrogen deficiency [6], and CD4+ helper T cells are the central organizers of adaptive immunity and various immunological diseases. Naive CD4+ T cells undergo functional differentiation into cytokine-secreting effector cells. The effector differentiation of helper T cells into the Th1, Th2, Th17, and regulatory T cells (Treg) subsets is determined by the cytokine environment [7,8]. Th17 cells among the T cell subtype play an important role in the induction of inflammation by producing pro-inflammatory cytokines, such as IL-17A and IL-17F [9]. On the other hand, Treg cells have an anti-inflammatory role and maintain self-tolerance by secreting cytokines, such as, transforming growth factor (TGF)-β and IL-10 [10]. IL-17 secreted by Th17 cells induces differentiation of osteoclast progenitors into mature osteoclasts *in vitro*. Moreover, treatment of human monocytes with only IL-17 induces osteoclastogenesis [11]. Whereas Th17 cells are key effector cells in diseases such as rheumatoid arthritis and osteoporosis, Treg cells are essential for dominant immunologic tolerance. Ovariectomy enhances Receptor activator of nuclear factor kappa-B ligand (RANKL), Tumor necrosis factor-α (TNF-α), and IL-17, and inhibition of these cytokines is likely to afford effective skeletal protection post-ovx [12].

The goal of osteoporosis therapy is to inhibit bone resorption by reducing osteoclastic production or activity. Hormone replacement therapies (HRT) based on, for example, estrogens, selective estrogen receptor modulators (SERMs), bisphosphonates, and calcitonin, inhibit bone loss. However, recent results of the Women’s Health Initiative demonstrated that women taking estrogen/progestin HRT are at elevated risk of breast cancer, coronary heart disease, and pulmonary embolism [13]. Therefore, an alternative treatment with fewer side effects is required for HRT.

The increasing interest shown in new and safer drugs from natural sources stems from safety concerns, and resulted in studies on the protective effects of alternative medicines on osteoporosis. Although many studies have been conducted on the treatment of osteoporosis, the problematic issue of side effects remains, and therefore, it would be useful to identify side-effect free natural compounds with a positive effect on osteoporosis [14].

*Alisma canaliculatum* (common name water plantain or “*taeksa*” in Korea) is commonly used in traditional Korean medicine [15]. This fruit has unique terpenoids, such as alisol A, B, and C; alisol A 24-acetate; alisol B 23-acetate; alisol C 23-acetate; alismalactone 23-acetate; alismols A, B, and C; sulfoorientalol A; oriediterpenol; and oriediterpenoside [16]. Furthermore, it has been shown to possess antibacterial [17], antitumor [18], and hepatoprotective [19] effects. In addition, alisol A-24 acetate from *Alisma canaliculatum* has been reported to inhibit osteoclast formation *in vitro* [20]. However, the antiosteoporotic effects of alisol A-24 acetate on postmenopausal osteoporosis have not been examined in ovariectomized (OVX) mice.

In the present study, we hypothesized that alisol A-24 acetate might prevent bone loss induced by estrogen deficiency, and in the present study examined its effects on bone deterioration in OVX mice.

## 2. Materials and Methods

The experimental protocol was approved by the Institutional Animal Care and Use Committee or Sunchon National University (permit number: SCNU IACUC-2015-05).

### 2.1. Isolation of Alisol A 24-Acetate from Alisma canaliculatum

**Figure 1:**
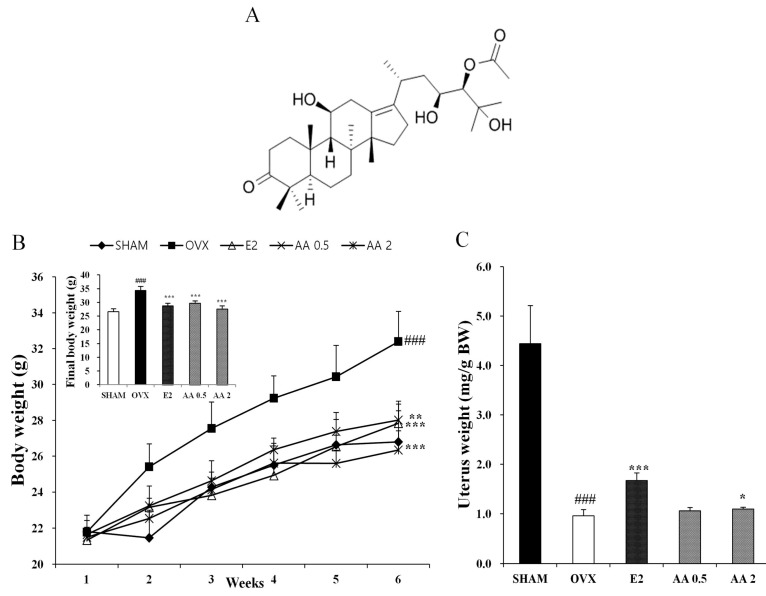
(**A**) Molecular structure of alisol A 24-acetate. Effect on (**B**) body weight and (**C**) uterine weight after six weeks treatment. Each value represents the mean ± SD for *n* = 5. ### *p* < 0.001, significantly different from sham mice. * *p* < 0.05, ** *p* < 0.01 and *** *p* < 0.001, significantly different from OVX (ovariectomy) mice.

Alisol A-24 acetate (AA; Figure 1A) prepared by Professor Nam Sang Jip at the Chemistry and Nano Science Department of Ewha Womans University (Seoul, Korea), as described previously [20], dissolved in dimethyl sulfoxide (DMSO) and diluted with distilled water immediately prior to use.

### 2.2. Animals and Experimental Treatments

Eight-week-old female C3H/HeN mice (weighing 20–22 g) were purchased from Orientbio (Orientbio Inc, Iksan, Korea). Animals were housed in standard polycarbonate cages under controlled conditions (22 ± 2 °C, RH 50%–60%, and a 12-h light/dark cycle) and allowed free access to commercial rodent chow (DAE-HAN Biolink, Daejeon, Korea) and water. In OVX animals, both ovaries (the primary source of endogenous estrogen) were removed under Zoletil-induced anesthesia. Animals were allowed to recover from surgery for 5 days prior to experiments. Mice were divided into 5 groups of 5 animals as follows: a sham-operated control group (a treatment naïve control group), which were administered water containing DMSO (dimethyl sulphoxide), i.p; a vehicle treated OVX group, which were also administered water containing DMSO, i.p; an OVX water-soluble β-estradiol (OVX E2 group; 0.03 μg/daily (s.c)) group as a positive control; and two OVX AA groups: a 0.5 μg OVX AA group and a 2 μg OVX AA group (animals were administered 0.5 or 2 μg/g BW (body weight) daily (i.p)). E2 and AA were administered for 6 weeks, and body weights were recorded weekly. At the end of the 6-week treatment period (15 weeks), animals were sacrificed by cervical dislocation. Serum was collected and stored at −80 °C until use, and the uteruses, spleens, thymuses, and tibias and femurs were removed and weighed. Femur and tibia lengths were measured using a Vernier caliper.

### 2.3. Measurements of Serum Ca, IP, and TCHO

Blood samples were maintained at room temperature for 1 h, and centrifuged at 5000 rpm for 5 min to obtain serum. Serum was separated immediately and stored at −80 °C. Serum calcium (Ca), inorganic phosphorus (IP), and total cholesterol (TCHO) levels were measured using a diagnostic slide kit and an automatic analyzer (Fuji Dri-Chem, Fuji, Japan).

### 2.4. Measurements of TRAP, E2 and BALP in Serum by ELISA

Tartrate-resistant acid phosphatase (TRAP) activity (a marker of bone resorption) and serum estradiol (E2) levels were measured using a TRAP enzyme-linked immunoassay (ELISA) kit (USCN Life Science, Wuhan, China) and an estradiol ELISA kit (Calbiotech, San Diego, CA, USA), respectively. Bone alkaline phosphatase (BALP) levels were measured using a BALP ELISA kit (Elabscience, Wuhan, China).

### 2.5. Flow Cytometry

To analyze intracellular cytokine levels, spleen cells were stimulated at 1 × 10^6^ cells/mL with 50 μg/mL of phorbolmyristate acetate (PMA) containing 1 uM innomycin for 5 h in the presence of 5 μg/mL of brefeline A for 3 h. These stimulated cells were stained with FITC-conjugated anti-CD4, APC-conjugated anti-CD25 (BD Biosciences; San Diego, CA, USA), and then fixed and permeabilized using PE-conjugated anti-IL-17A or forkhead box P3 (Foxp3) (BD Biosciences; San Diego, CA, USA). All data were analyzed using FACScantoII (BD Bioscience).

### 2.6. Bone Structure Analysis

Bone morphometric parameters of femurs (cleaned of adherent soft tissues) were assessed using a micro-computed tomography (micro-CT) system (Skyscan 1172, Kontich, Belgium). Scans were taken at a source voltage of 49 kV and a source current of 200 μA. The resolution was set at 17.09 μm and the rotation step at 0.4°. 2D and 3D images were obtained for visualization and display. The structural parameters for trabecular bone were analyzed using CTAn software (Skyscan). Bone volume densities (BV/TV), bone surface/total volum (BS/TV), bone surface/bone volume (BS/BV), trabecular thickness/separation/number/pattern factor (Tb.Th, Tb.Sp, Tb.N, and Tb.Pf, respectively) values, structure model indices (SMIs), and bone mineral density (BMDs) of femurs were calculated. The distal femur metaphysis was used as a region of interest for the analysis.

### 2.7. Histological Analysis

Femurs were fixed in 4% paraformaldehyde, decalcified in 10% EDTA, dehydrated, embedded in paraffin, sectioned at 5 μm, and stained with hematoxylin and eosin (H & E). The femoral regions studied were; the secondary spongiosa, the trabecular portion of the distal femur, 12 mm distal to the epiphyseal plate and extending to 6 mm. Sections (7 mm) were deparaffinized in 2-ethoxyethyl acetate and stained with Masson’s trichrome.

### 2.8. Statistical Analysis

Results are presented as the means ± SDs. The significances of differences were analyzed using the Student’s *t*-test. Probability values of less than 0.05 were considered significant.

## 3. Results and Discussion

### 3.1. Effects of AA on Body, Uterus, and Bone Weights in OVX Mice

As shown in Figure 1B, mice in all five experimental groups had similar initial body weights. At six weeks after surgery, the OVX group showed a significant increase in final body weight as compared with the SHAM group (*p* < 0.001). Treatment with AA resulted in a significant reduction in OVX-induced weight gain in OVX mice at 0.5 and 2 μg/g daily (*p* < 0.001). Uterine weights of all OVX mice were significantly lower than in the SHAM group (*p* < 0.001), which confirmed the success of the surgical procedure, and mice in the OVX groups exhibited atrophy of uterine tissue. Uterine weights in the AA 2 μg/g group were significantly higher than in the OVX group (Figure 1C).

**Table 1:** Effect on AA (alisol A-24 acetate) on weight and length in bone of OVX mice.

	Length (mm)		Weight (mg)	
	Tibia	Femur	Tibia	Femur
SHAM	19.528 ± 0.606	15.986 ± 0.074	45.9 ± 3.937	59.68 ± 2.791
OVX	18.338 ± 0.638 #	15.538 ± 0.349 #	40.3 ± 4.702	51.48 ± 4.827 ##
E2	18.784 ± 0.071	16.344 ± 0.129 **	45.26 ± 1.024	57.84 ± 1.44 *
AA 0.5	18.556 ± 0.099	16.18 ± 0.154 **	43.9 ± 2.747	56.32 ± 2.681
AA 2	18.592 ± 0.112	15.826 ± 0.119	47.22 ± 2.734 *	58.84 ± 2.855 *

Each value represents the mean ± SD for *n* = 5. # *p* < 0.05 and ## *p* < 0.01 significantly different from sham mice. * *p* < 0.05 and ** *p* < 0.01 significantly different from OVX mice.

In a subsequent experiment, the effects of AA on bone weight and length were evaluated. Femur weights in the OVX control group were significantly lower than in the SHAM group. The femur weights of OVX mice were suppressed in reduction by treatment with AA 2 μg/g (*p* < 0.05). Femur and tibia lengths were lower in the OVX group than in the SHAM group, and femur lengths were significantly greater in the AA 0.5 μg/g group than in the OVX group. Supplementation with AA at 0.5 and 2 μg/g positively affected femur and tibia weights and lengths as compared with the OVX group (Table 1).

### 3.2. Effects of AA on the Balance between Th17 and Regulatory T Cell

**Figure 2:**
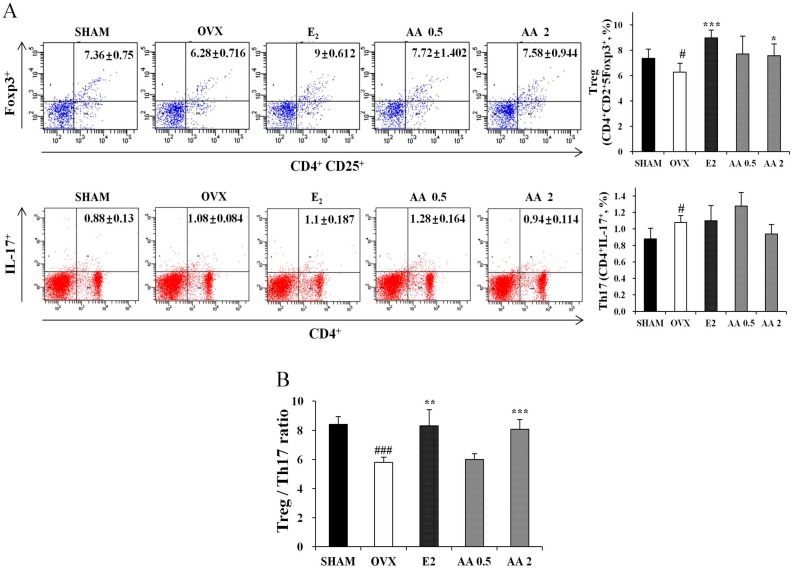
AA treatment increase Treg cells and decrease Th17 cell. (**A**) After the isolation of splenocytes from AA treated mice or vehicle treated mice, the populations of IL-17 producing CD4+ T cells, and Foxp3 producing C25+ T cells were analyzed using antibodies specific for CD4, CD25, Foxp3 and IL-17 by intracellular flow cytometric analysis. (**B**) Treg/Th17 ratio. Each value represents the mean ± SD for *n* = 5. # *p* < 0.05 and ### *p* < 0.001, significantly different from sham mice. * *p* < 0.05, ** *p* < 0.01 and *** *p* < 0.001, significantly different from OVX mice.

We next investigated whether AA affects the population of new Th cells, that is, Th17 cells and regulatory T cells. Th17 cells and regulatory T cell numbers were determined using IL-17A or Foxp3 as markers by flow cytometry. Mouse splenocytes obtained from animals in AA 2 μg/g groups showed slightly lower cytokine expression of Th17 cells related factors such as IL-17A. However, it is not a statistically significant difference. Nevertheless, AA significantly increased numbers of CD4+CD25+Foxp3+ regulatory T cells in spleen tissues as compared with OVX group (*p* < 0.05) (Figure 2A). As shown in Figure 2B, the ratio of Th17 cell numbers to regulatory T cell numbers was significantly decreased by AA in OVX mice.

### 3.3. Effects of AA on Serum Biochemical Markers (Ca and IP) and TCHO

The effects of six weeks of treatment on serum biochemical parameters (Ca and IP) and TCHO are shown in Table 2. The level of serum calcium was significantly lower in the AA-treated groups (0.5 μg/g and 2 μg/g) than in the OVX group (*p* < 005 and *p* < 0.01). The level of phosphorus was higher in the OVX group than in the SHAM group (*p* < 0.05), and was slightly lower in the AA treated groups than in OVX group. The total serum cholesterol (TCHO) was higher in the OVX group than in the SHAM group (*p* < 0.001), and was significantly lower in the AA 0.5 and 2 μg/g groups than in OVX group (*p* < 0.05 and *p* < 0.01, respectively).

**Table 2:** Effect of AA on serum biochemical markers.

	SHAM	OVX	E2	AA 0.5	AA 2
Calcium (mg/dl)	10.32 ± 0.303	10.8 ± 0.173 #	10.38 ± 0.277 *	10.4 ± 0.212 *	10.22 ± 0.205 **
Phosphorus (mg/dl)	5.22 ± 0.507	7.7 ± 1.632 #	7.22 ± 0.963	6.86 ± 1.033	7.32 ± 1.094
Total cholesterol (mg/dl)	118.8 ± 9.834	197.4 ± 6.656 ###	156.6 ± 5.459 ***	177.8 ± 11.432 *	162.2 ± 14.533 **

Each value represents the mean ± SD for *n* = 5. # *p* < 0.05 and ### *p* < 0.001 significantly different from sham mice. * *p* < 0.05, ** *p* < 0.01 and *** *p* < 0.001 significantly different from OVX mice.

### 3.4. Effects of AA on Serum TRAP, E2, and BALP

**Figure 3:**
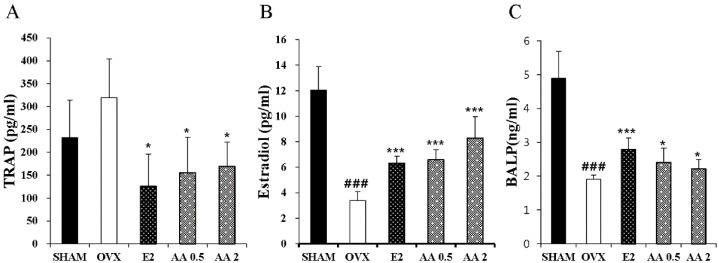
Effect of AA on serum TRAP, Estradiol, and BALP. In control, SHAM -operated mice and OVX mice with or without the injection of AA (0.5 μg/g, 2 μg/g/day, I.P) for six weeks, Serum (**A**) TRAP and (**B**) Estradiol were measured by ELISA kit. Each value represents the mean ± SD for *n* = 4~5. ### *p* < 0.001, significantly different from sham mice. * *p* < 0.05 and *** *p* < 0.001 significantly different from OVX mice.

To evaluate the effect of AA treatment on bone turnover in OVX mice, we measured serum estradiol, tartrate-resistant acid phosphatase (TRAP), and bone specific alkaline phosphatase (BALP) levels. TRAP activity was slightly higher in the OVX group than in the sham group, and significantly lower in the AA 0.5 and 2 μg/g groups than in the OVX group (*p* < 0.05 and *p* < 0.05, respectively) (Figure 3A). Serum estradiol was significantly lower in the OVX group than in the SHAM group (*p* < 0.001), and the 0.5 and 2.0 μg/g AA groups had significantly higher estradiol levels than the OVX group (*p* < 0.001) (Figure 3B). Furthermore, BALP (an osteoblast activity marker) were significantly higher in the AA 0.5 and 2 μg/g groups than in the OVX group (*p* < 0.05 and *p* < 0.001) (Figure 3C).

### 3.5. Effects of AA on Bone Microarchitecture

**Figure 4:**
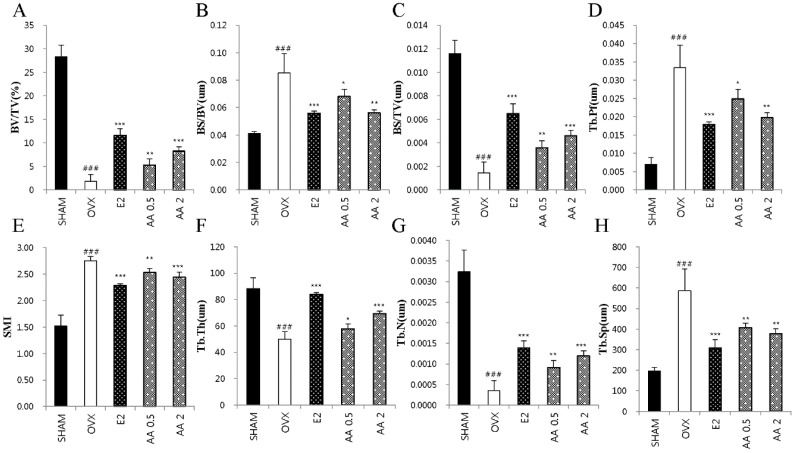
Effect of AA on trabecular morphometric parameters in distal femur of OVX mice. Mice were treated with vehicle, AA (0.5, 2 μg/g/day, I.P) for 6 weeks. (**A**) Bone volume/tissue volume (BV/TV); (**B**) bone surface/bone volume (BS/BV); (**C**) bone surface/tissue volume (BS/TV); (**D**) trabecular pattern factor (Tb.Pf); (**E**) structure model index (SMI); (**F**) trabecular thickness (Tb.Th); (**G**) trabecular number (Tb.N); and (**H**) trabecular separation (Tb.Sp) as analyzed with micro-CT Skyscan CTAn software. Each value represents the mean ± SD for *n* = 5. ### *p* < 0.001, significantly different from sham mice. * *p* < 0.05, ** *p* < 0.01 and *** *p* < 0.001 significantly different from OVX mice.

**Figure 5:**
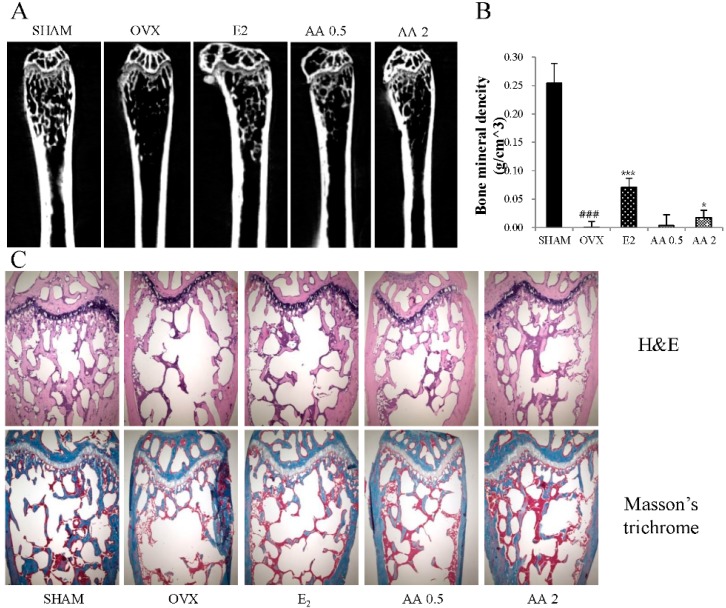
The effect of treatment with AA (alisol A-24 acetate) on the femur trabecular microarchitecture in OVX (ovaritectomy) mice: (**A**) two dimensional micro-computed tomography (micro-CT) images of the femoral trabecular bone; (**B**) bone mineral density (BMD) was assessed by micro-CT; and (**C**) histological analysis of femur with H & E and Masson’s trichrome staining. Magnification: 40-fold. Each value represents the mean ± SD for *n* = 5. ### *p* < 0.001, significantly different from sham mice. * *p* < 0.05 and *** *p* < 0.001 significantly different from OVX mice.

As shown in Figure 4, OVX altered femoral trabecular architecture, but E2 and AA treatment reduced this OVX-induced alteration. The OVX group exhibited significant changes in bone volume density (BV/TV), bone surface/bone volume (BS/BV), bone surface density (BS/TV), trabecular pattern factor (Tb.Pf), structure model index (SMI), trabecular thickness (Tb.Th), trabecular number (Tb.N), and trabecular separation (Tb.Sp) *vs.* the SHAM group, suggesting that OVX caused significant loss of trabecular bone. AA treatment significantly increased BV/TV, BS/TV, and Tb.N at doses of 0.5 and 2 μg/g *vs.* OVX mice (*p* < 0.01 and *p* < 0.001, respectively) (Figure 4A,C,G), and significantly increased Tb.Th at 0.5 and 2 μg/g (*p* < 0.05 and *p* < 0.001, respectively) (Figure 4F). In contrast, BS/BV, Tb.Pf, SMI, and Tb.Sp were higher in the OVX group than in the SHAM group (*p* < 0.001). Treatment with AA at 0.5 or 2.0 μg/g decreased BS/BV and Tb.Pf *vs.* OVX group (*p* < 0.05 and *p* < 0.01) (Figure 4B,D, respectively), as were SMI and Tb.Sp (*p* < 0.01 and *p* < 0.001, respectively) (Figure 4E,H). To determine the effect of AA on OVX-induced trabecular bone deterioration, bone mineral densities (BMD) were analyzed by micro-CT. The results obtained showed that AA at 0.5 and 2 μg/g prevented femoral bone loss in OVX mice (Figure 5A). Mean BMD in the OVX group was lower than in the SHAM group (*p* < 0.001), but was higher in the 2 μg/g AA group than in the OVX group (*p* < 0.05) (Figure 5B).

### 3.6. Discussion

This study shows treatment with alisol A-24 acetate (AA) from *Alisma canaliculatum* reduced bone resorption in ovariectomized (OVX) mice. In our mouse model, OVX-induced osteoporosis led to bone loss, and AA treatments effectively prevented loss of trabecular bone and improved trabecular bone microstructure. Furthermore, the levels of markers bone turnover, that is, estradiol, TRAP, and BALP, were improved in AA treated mice. The decrease in Treg/Th17 ratio caused by OVX was suppressed in mice treated with AA. These results suggest that AA has a protective effect on OVX-induced bone loss associated with changes in bone turnover markers and Treg/Th17 ratio.

Osteoporosis poses a substantial public health problem, and imposes a tremendous burden on patients and society [21]. Many women are still prescribed hormone replacement therapy (HRT) for post-menopausal symptoms. However, the prolonged use of HRT has been associated with breast cancer, venous thromboembolism, coronary heart disease, and stroke [22]. Therefore, alternative, safe, effective medicines are required that are free of the side effects on HRT, and for this reason efforts are being made to explore the effects of natural compounds, especially those of plant origin [23].

The OVX mice are considered an excellent model for studying osteoporosis in post-menopausal women [24]. Other experimental studies have reported that body weights are higher in OVX mice due to increases in fatty deposits [25]. In the present study, AA was found to suppress OVX-induced increases in body weights. Furthermore, dramatic decreases in uterine weights evidence effectiveness of OVX for reducing estrogen levels [26]. In the present study, OVX-induced atrophy of the uterus was reduced by the administration of AA. Some authors have reported femur weights in OVX mice are lower than in sham operated mice, and it has been suggested that AA influences interaction between estrogen and its receptor [27]. In the present study, the decrease in femur weight induced by OVX was suppressed by AA.

Bone loss in postmenopausal women may also be prevented by estradiol treatment as it suppresses bone cell activity [28]. This finding stimulated the development of biochemical markers to assist in the monitoring of treatment efficiency. Biochemical markers of bone turnover are products release from osteoblasts and osteoclasts [29]. Bone specific alkaline phosphatase (BALP), which is associated with osteoblast activity, has been used to assess bone formation. For example, BALP activity was found to be significantly lower in OVX rats than in sham controls [30]. Furthermore, Su *et al.*, found tartrate-resistant acid phosphatase (TRAP; a bone resorption marker) levels were higher in an OVX group [31]. In the present study, treatment with AA increased BALP and suppressed TRAP activity in OVX mice, suggesting AA effectively prevented bone loss due to estrogen deficiency. Estrogen plays an important role in the maintenance of bone mass, and its serum levels are reduced by OVX [32,33]. Furthermore, estrogen prevents excessive release of calcium from the body. For these reasons, estrogen and calcium supplementation is appropriate when treating osteoporosis [34]. In the present study, OVX-induced estradiol reduction was inhibited by AA, and calcium levels in OVX mice treated with AA remained at the sham group level. Also, we measured the level of TNF-α in serum. As a result, there was no significant difference between the sham group (0.196 ± 0.004) and OVX group (0.196 ± 0.012). The TNF-α was significantly lower in the AA 0.5 μg/g (0.167 ± 0.01, *p* < 0.01)) and 2 μg/g (0.168 ± 0.006, *p* < 0.01) treated group than in the OVX group (Data not shown).

Some authors have reported that T cells play a key role in the bone loss associated with estrogen deficiency. Th17 cells produce IL-17, which is an important mediator of inflammatory bone diseases, and Il-17 mediated osteoclast differentiation in bone marrow cells and was found to be suppressed by estradiol. In contrast to Th17 cells, Treg cells may be the most important immune system regulator. Treg cells suppress bone resorption by secreting transforming growth factor-beta 1 (TGF-β1) and IL-10. Some have reported diminished Treg cell numbers in OVX mice, and thus, it has been suggested Treg cells offer a therapeutic target for the treatment of bone loss in OVX mice [35,36]. In the present study, treatment with AA enhanced Treg levels and Treg/Th17 ratios in OVX mice, which suggests AA reduced bone loss in OVX mice ovariectomy is associated with the differentiation of Treg cells.

The clinically measurable properties of bone that have been shown to independently predict a future osteoporotic fracture are bone mineral density (BMD), and bone microarchitecture, that is, trabecular bone volume, number, and thickness [37]. Ovariectomy is typically linked with deteriorations in trabecular structure and BMD [38]. The present study shows that AA has a positive effect on trabecular morphometric parameters, that is, bone volume density (BV/TV), thickness, number, separation, and BMD in OVX mice. Furthermore, 2D images and histological analysis showed that AA protects against bone loss in OVX mice. These results suggest that AA has inhibitory effect on OVZ-induced bone loss and deterioration.

## 4. Conclusions

In summary, we provide evidence that six weeks of 0.5 or 2 μg/g of AA administration to osteoporotic mice suppresses body weight increases and uterine weight reductions, and improves bone biochemical markers, such as, Ca, TCHO, BALP, estradiol, and TRAP levels and Treg/Th17 ratios. Furthermore, AA administration improved the femoral BMD and trabecular microstructure. We believe that alisol A-24 acetate from *Alisma canaliculatum* has potential for further development as a natural alternative for the management of postmenopausal osteoporosis.

## References

[1] Raggatt L.J., Partridge N.C. (2010). Cellular and molecular mechanisms of bone remodeling. J. Biol. Chem..

[2] Tseng S.H., Sung C.H., Chen L.G., Lai Y.J., Chang W.S., Sung H.C., Wang C.C. (2014). Comparison of chemical compositions and osteoprotective effects of different sections of velvet antler. J. Ethnopharmacol..

[3] Tantikanlayaporn D., Wichit P., Weerachayaphorn J., Chairoungdua A., Chuncharunee A., Suksamrarn A., Piyachaturawat P. (2013). Bone sparing effect of a novel phytoestrogen diarylheptanoid from Curcuma comosa Roxb. in ovariectomized rats. PLoS ONE.

[4] Raisz L.G. (2005). Pathogenesis of osteoporosis: Concepts, conflicts, and prospects. J. Clin. Investig..

[5] Lerner U.H. (2006). Bone remodeling in post-menopausal osteoporosis. J. Dent. Res..

[6] Tyagi A.M., Srivastava K., Mansoori M.N., Trivedi R., Chattopadhyay N., Singh D. (2012). Estrogen deficiency induces the differentiation of IL-17 secreting Th17 cells: A new candidate in the pathogenesis of osteoporosis. PLoS ONE.

[7] Zhu J., Paul W.E. (2010). Heterogeneity and plasticity of T helper cells. Cell Res..

[8] Yang X.O., Zhang H., Kim B.S., Niu X., Peng J., Chen Y., Kerketta R., Lee Y.H., Chang S.H., Corry D.B. (2013). The signaling suppressor CIS controls proallergic T cell development and allergic airway inflammation. Nat. Immunol..

[9] Bedoya S.K., Lam B., Lau K., Larkin J. (2013). Th17 cells in immunity and autoimmunity. Clin. Dev. Immunol..

[10] Afzali B., Lombardi G., Lechler R.I., Lord G.M. (2007). The role of T helper 17 (Th17) and regulatory T cells (Treg) in human organ transplantation and autoimmune disease. Clin. Exp. Immunol..

[11] Alves C.H., Farrell E., Vis M., Colin E.M., Lubberts E. (2015). Animal Models of Bone Loss in Inflammatory Arthritis: From Cytokines in the Bench to Novel Treatments for Bone Loss in the Bedside-a Comprehensive Review. Clin. Rev. Allergy Immunol..

[12] Tyagi A.M., Mansoori M.N., Srivastava K., Khan M.P., Kureel J., Dixit M., Shukla P., Trivedi R., Chattopadhyay N., Singh D. (2014). Enhanced immunoprotective effects by anti-IL-17 antibody translates to improved skeletal parameters under estrogen deficiency compared with anti-RANKL and anti-TNF-α antibodies. J. Bone Miner. Res..

[13] Downey P.A., Siegel M.I. (2006). Bone biology and the clinical implications for osteoporosis. Phys. Ther..

[14] Lee J.W., Jhee O., Yuan H., Kim T., Kim D., Lee M., Om A., Lee B., Park S.K., Kang J. (2005). Effect of Korean oriental medicine extract on bone mass as compared with alendronate in ovariectomized rats. J. Med. Food..

[15] Hossain M.E., Kim G.M., Lee S.K., Yang C.J. (2012). Growth performance, meat yield, oxidative stability, and Fatty Acid composition of meat from broilers fed diets supplemented with a medicinal plant and probiotics. Asian-Australas. J. Anim. Sci..

[16] Hossain M.E., Ko S.Y., Kim G.M., Firman J.D., Yang C.J. (2012). Evaluation of probiotic strains for development of fermented *Alisma canaliculatum* and their effects on broiler chickens. Poult. Sci..

[17] Mikamo H., Kawazoe K., Izumi K., Sato Y., Tamaya T. (1998). Effects of crude herbal ingredients on intrauterine infection in a rat model. Curr. Ther. Res..

[18] Huang Y.T., Huang D.M., Chueh S.C., Teng C.M., Guh J.H. (2006). Alisol B acetate, a triterpene from Alismatis rhizoma, induces Bax nuclear translocation and apoptosis in human hormone-resistant prostate cancer PC-3 cells. Cancer Lett..

[19] Jang M.K., Han Y.R., Nam J.S., Han C.W., Kim B.J., Jeong H.S., Ha K.T., Jung M.H. (2015). Protective Effects of Alisma orientale Extract against Hepatic Steatosis via Inhibition of Endoplasmic Reticulum Stress. Int. J. Mol. Sci..

[20] Kim K.J., Leutou A.S., Yeon J.T., Choi S.W., Kim S.H., Yee S.T., Choi K.H., Nam S.J., Son Y.J. (2015). The Inhibitory Effect of Alisol A 24-Acetate from *Alisma canaliculatum* on Osteoclastogenesis. Int. J. Endocrinol..

[21] Ray N.F., Chan J.K., Thamer M., Melton L.J. (1997). Medical expenditures for the treatment of osteoporotic fractures in the United States in 1995: Report from the National Osteoporosis Foundation. J. Bone Miner. Res..

[22] Canonico M., Plu-Bureau G., Lowe G.D., Scarabin P.Y. (2008). Hormone replacement therapy and risk of venous thromboembolism in postmenopausal women: Systematic review and meta-analysis. BMJ.

[23] Liu Z.G., Zhang R., Li C., Ma X., Liu L., Wang J.P., Mei Q.B. (2009). The osteoprotective effect of Radix Dipsaci extract in ovariectomized rats. J. Ethnopharmacol..

[24] Wronski T.J., Dann L.M., Qi H., Yen C.F. (1993). Skeletal effects of withdrawal of estrogen and diphosphonate treatment in ovariectomized rats. Calcif. Tissue Int..

[25] Notomi T., Okimoto N., Okazaki Y., Nakamura T., Suzuki M. (2003). Tower climbing exercise started 3 months after ovariectomy recovers bone strength of the femur and lumbar vertebrae in aged osteopenic rats. J. Bone Miner. Res..

[26] Hidaka S., Okamoto Y., Nakajima K., Suekawa M., Liu S.Y. (1997). Preventive effects of traditional Chinese (Kampo) medicines on experimental osteoporosis induced by ovariectomy in rats. Calcif. Tissue Int..

[27] Park J.A., Ha S.K., Kang T.H., Oh M.S., Cho M.H., Lee S.Y., Park J.H., Kim S.Y. (2008). Protective effect of apigenin on ovariectomy-induced bone loss in rats. Life Sci..

[28] Sims N.A., Morris H.A., Moore R.J., Durbridge T.C. (1996). Estradiol treatment transiently increases trabecular bone volume in ovariectomized rats. Bone.

[29] Swaminathan R. (2001). Biochemical markers of bone turnover. Clin. Chim. Acta..

[30] Fahmy S.R., Soliman A.M., Sayed A.A., Marzouk M. (2015). Possible antiosteoporotic mechanism of Cicer arietinum extract in ovariectomized rats. Int. J. Clin. Exp. Pathol..

[31] Su S.J., Yeh Y.T., Shyu H.W. (2013). The preventive effect of biochanin A on bone loss in ovariectomized rats: Involvement in regulation of growth and activity of osteoblasts and osteoclasts. Evid. Based Complement. Altern. Med..

[32] Mohamed M.K., Abdel-Rahman A.A. (2000). Effect of long-term ovariectomy and estrogen replacement on the expression of estrogen receptor gene in female rats. Eur. J. Endocrinol..

[33] Lim D.W., Lee Y., Kim Y.T. (2014). Preventive effects of Citrus unshiu peel extracts on bone and lipid metabolism in OVX rats. Molecules.

[34] Davis J.W., Ross P.D., Johnson N.E., Wasnich R.D. (1995). Estrogen and calcium supplement use among Japanese-American women: Effects upon bone loss when used singly and in combination. Bone.

[35] Lai N., Zhang Z., Wang B., Miao X., Guo Y., Yao C., Wang Z., Wang L., Ma R., Li X. (2015). Regulatory effect of traditional Chinese medicinal formula Zuo-Gui-Wan on the Th17/Treg paradigm in mice with bone loss induced by estrogen deficiency. J. Ethnopharmacol..

[36] Liu J.C., Zhou C.H., Zhang X., Chen Y., Xu B.L., Cui L., Xu D.H. (2014). Effect of 1,25-dihydroxyvitamin D_3_ on regulatory T cells in ovariectomized mice. Biomed. Environ. Sci..

[37] Recker R., Masarachia P., Santora A., Howard T., Chavassieux P., Arlot M., Rodan G., Wehren L., Kimmel D. (2005). Trabecular bone microarchitecture after alendronate treatment of osteoporotic women. Curr. Med. Res. Opin..

[38] Huang G., Wu J., Wang S., Wei Y., Chen F., Chen J., Shi J., Xia J. (2015). Pycnogenol^®^ treatment inhibits bone mineral density loss and trabecular deterioration in ovariectomized rats. Int. J. Clin. Exp. Med..

